# A quantitative proteomic screen of the *Campylobacter jejuni* flagellar-dependent secretome

**DOI:** 10.1016/j.jprot.2016.11.009

**Published:** 2017-01-30

**Authors:** Eoin Scanlan, Lu Yu, Duncan Maskell, Jyoti Choudhary, Andrew Grant

**Affiliations:** a Department of Veterinary Medicine, University of Cambridge, Madingley Road, Cambridge CB3 0ES, United Kingdom; b Proteomic Mass Spectrometry, Wellcome Trust Sanger Institute, Hinxton CB10 1SA, United Kingdom

**Keywords:** *Campylobacter jejuni*, Secretome, Type III secretion system, Flagella, SILAC, Mass spec

## Abstract

*Campylobacter jejuni* is the leading cause of bacterial gastroenteritis in the world. A number of factors are believed to contribute to the ability of *C. jejuni* to cause disease within the human host including the secretion of non-flagellar proteins *via* the flagellar type III secretion system (FT3SS). Here for the first time we have utilised quantitative proteomics using stable isotope labelling by amino acids in cell culture (SILAC), and label-free liquid chromatography-mass spectrometry (LC/MS), to compare supernatant samples from *C. jejuni* M1 wild type and flagella-deficient (*flgG* mutant) strains to identify putative novel proteins secreted *via* the FT3SS. Genes encoding proteins that were candidates for flagellar secretion, derived from the LC/MS and SILAC datasets, were deleted. Infection of human CACO-2 tissue culture cells using these mutants resulted in the identification of novel genes required for interactions with these cells. This work has shown for the first time that both CJM1_0791 and CJM1_0395 are dependent on the flagellum for their presence in supernatants from *C. jejuni* stains M1 and 81–176.

**Biological significance:**

This study provides the most complete description of the *Campylobac er jejuni* secretome to date. SILAC and label-free proteomics comparing mutants with or without flagella have resulted in the identification of two *C. jejuni* proteins that are dependent on flagella for their export from the bacterial cell.

## Highlights

• Catalogue of the *Campylobacter jejuni* M1 flagella-dependant secretome
• Importance of multiple *C. jejuni* M1 proteins for interaction with CACO-2 cells
• CJM1_0791 and CJM1_0395 require flagellum for presence in supernatant.

## Graphical abstract

**Figure:**
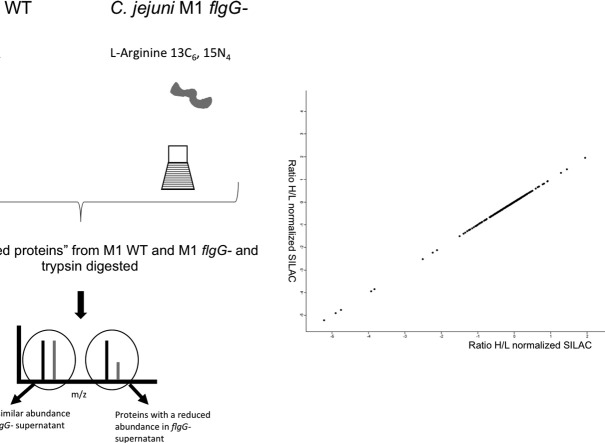

## 1 Introduction

*Campylobacter jejuni* is the leading cause of foodborne bacterial gastroenteritis in the world [1]. Cases of *C. jejuni* infection are most commonly acute and self-limiting in healthy individuals, however a number of complications can occur post-infection. The most serious of these is the development of Guillian-Barré syndrome, an acute demyelinating disease resulting in progressive ascending paralysis [2].

Research investigating *C. jejuni* pathogenesis has identified important roles for flagellum-dependent motility, adhesion/invasion of host epithelial cells and toxin production among others, as factors important for causing human disease [3]. Although these factors are frequently observed among bacterial pathogens, *C. jejuni* appears unlike other enteric pathogens with respect to extracellular protein secretion [4].

It has been proposed that *C. jejuni* utilises its flagellum not only for motility but also to act as a conduit for the secretion of non-flagellar proteins [5]. Previous studies have identified multiple components of the *C. jejuni* flagellum that are required for the export of the *Campylobacter* invasion antigens (Cia), and other non-flagellar proteins, some of which have been implicated in the ability of *C. jejuni* to invade human intestinal cell lines [6], [7], [8], [9], [10], [11], [12], [13], [14], [15], [16], [17]. CiaB was the first non-flagellar *C. jejuni* protein proposed to be dependent upon the flagellum for secretion, and is required for efficient invasion of INT-407 cells [9]. CiaB is also suggested to be required for the secretion of at least two other proteins, CiaC and CiaI [7], [9], [11], [12]. CiaC and CiaI secretion requires a minimum flagellar structure containing the hook protein FlgE [7], [11], [12]. CiaC is necessary for wild type invasion of INT-407 cells [7]. A *ciaI* mutant of *C. jejuni* F38011 displays reduced survival within INT-407 cells, while a *ciaI* mutant of *C. jejuni* 81–176 is reduced in its ability to colonize the chicken intestinal tract [11], [12]. Another Cia protein that is dependent on the flagellum for secretion, CiaD, is also required for maximal invasion of INT-407 cells [13], [14]. Furthermore, FlaC and FspA also require a minimum flagellar structure for extracellular secretion [15], [16]. FlaC, which has high sequence similarity to the major and minor flagellin filaments of *C. jejuni*, binds Hep-2 cells, and a *C. jejuni* TGH9011 *flaC* mutant is reduced in its ability to invade those cells [15]. FspA is readily observed as two isoforms among different *C. jejuni* isolates, and the external addition of FspA2 induces apoptosis of INT-407 cells [16]. Another study has identified a group of proteins dependent on σ^28^ for their production and secretion and hence that are expressed under the same conditions as *flaA*, resulting in these being annotated as the Feds proteins (flagellar co-expressed determinants). This group of four proteins is required for colonization of chickens, with FedA also important for invasion of human-derived T84 cells [12].

Although multiple *C. jejuni* proteins are dependent on the flagellum for their secretion, a possible mechanism by which it might interact with host cells has yet to be described. Moreover, the ability of a *C. jejuni* strain 81–176 *ciaB* mutant to invade T84 cells is not statistically different from the wild type [17]. Much of the literature describing non-flagellar protein secretion *via* the *C. jejuni* flagellum has documented proteins secreted to the extracellular environment. The biological relevance of this for effector-like proteins is unclear, as they are likely to be subjected to degradation by host proteases. There has also been no identification of a conserved amino acid sequence present among non-flagellar *C. jejuni* proteins that might act as a flagellar secretion signal, as has been described for the *Yersinia enterocolitica* protein YplA [18].

In this study we have used a combination of SILAC (stable isotope labelling by amino acids in cell culture) and label-free LC-MS (liquid chromatography-mass spectrometry) to investigate the *C. jejuni* flagellum-dependant secretome. This has enabled a comprehensive screen of the *C. jejuni* secretome, in an attempt to identify previously undescribed proteins, both flagellar and non-flagellar, being transported *via* the *C. jejuni* flagellar type III secretion apparatus (FT3SS). Utilizing *C. jejuni* stain M1 is a suitable strain for this purpose as it has been documented to colonize both human and avian hosts [19]. Therefore, in using strain M1 we hope to comprehensively assess flagella-dependent proteins, possibly contributing to colonization of chickens and/or the development of human disease.

## 2 Materials and methods

### 2.1 Bacterial strains and culture conditions

All wild type strains and defined mutants are described in Table S1. *C. jejuni* strains were routinely cultured on Brain Heart Infusion (BHI, Oxoid) agar plates supplemented with 5% defibrinated horse blood (Oxoid) and 5 μg/ml trimethoprim (TrM). Strains containing FLAG-tagged proteins were grown in the presence of 50 μg/ml kanamycin (Km). Gene deletion mutants were grown in the presence of 10 μg/ml chloramphenicol (Cm). FLAG-tagged strains also containing gene deletions were grown in the presence of 50 μg/ml Km and 10 μg/ml Cm. Microaerophilic conditions for *C. jejuni* growth (5% O_2_, 10% CO_2_ and 85% N_2_) were maintained by a MACS VA500 variable atmosphere work station (Don Whitley Scientific).

### 2.2 Isotopic labelling of *C. jejuni* cultures

*C. jejuni* strain M1 and its isogenic *flgG* mutant were grown on 1% SILAC DMEM plates supplemented with 10 mM l-Glutamine (Sigma) and either l-Arginine-HCL (Thermo Fisher Scientific) or l-Arginine ^13^C_6_, ^15^N_4_ (Thermo Fisher Scientific). Powdered SILAC DMEM (Thermo Fisher Scientific) was dissolved in water to create a 2 × SILAC DMEM solution. Amounts of either l-Arginine-HCL or l-Arginine ^13^C_6_, ^15^N_4_ were added to the solution, dissolved, and passed through a 0.22 μm filter. To this, an equal volume of sterile 2% select agar (Sigma) was added and supplemented with 10 mM l-Glutamine. For validation of the SILAC data by Western immunoblotting, 1% DMEM plates were composed of 2 × standard DMEM (Millipore) mixed with 2% sterile agar as above, and supplemented with 10 mM l-Glutamine. Bacterial strains were streaked on relevant media from frozen stocks and incubated at 42 °C under microaerophilic conditions for 48 h.

### 2.3 Preparation of *C. jejuni* supernatants

For SILAC labelled cultures, once isotopic amino acid incorporation was achieved a previously published protocol for the generation of *C. jejuni* supernatants [6], [12] was utilised, adapted here for use with DMEM. Bacteria were suspended to an OD_600nm_ of 0.6 in 20mls SILAC DMEM (Thermo Fisher Scientific) supplemented with a relevant amount of “light” (wild type samples) or “heavy” (mutant samples) l-Arginine, and 10 mM l-Glutamine. This 20 ml culture was overlaid onto 5 ml 1% SILAC DMEM agar and was incubated statically at 42 °C under microaerophilic conditions for 4 h. Various growth experiments revealed that these conditions were optimal for *C. jejuni* growth in DMEM (data not shown). For the M1 *flgG* mutant, chloramphenicol was added at a concentration of 10 μg/ml to both 1% DMEM agar and liquid SILAC DMEM. At the end of the incubation period, OD_600nm_ measurements were taken for each culture, and 1 ml of each culture was pelleted for subsequent whole cell protein sample preparation. Each remaining 18 ml culture was centrifuged at 4000 × g for 20 min, the supernatant was transferred to a fresh tube and the centrifugation step was repeated. Supernatants were then passed through a syringe with a 0.22 μm filter to remove any remaining whole bacteria. Following this, 15 ml of each supernatant was transferred to an Amicon Ultra centrifugal filter unit (Millipore) and centrifuged at 4000 g for 30 min. To make supernatant samples for SILAC validation experiments, the above protocol was followed using 1% standard DMEM agar (Gibco) supplemented with 10 mM l-Glutamine. Concentrated supernatant samples were divided into aliquots which were stored at − 20 °C for future use.

### 2.4 LC-MS/MS analysis

The unlabelled, or SILAC labelled samples were reduced with tris(2-carboxyethyl) phosphine (TCEP) then alkylated with iodoacetamide (Sigma) followed by digestion by trypsin (Thermo Fisher Scientific) overnight at 37 °C. 0.5 μg (unlabelled samples) or 1.5 μg (SILAC samples) of the digest were submitted for the nano LC-MS/MS analyses on an Ultimate 3000 RSLCnano System coupled to a LTQ Orbitrap Velos hybrid mass spectrometer equipped with a nanospray source. The peptides were first loaded and desalted on a PepMap C18 trap column (100 μm id × 20 mm, 5 μm) then separated on a PepMap C18 analytical column (75 μm id × 500 mm, 2 μm) over a 90 min (unlabelled samples) or 180 min (SILAC labelled samples) linear gradient of 4–32% CH_3_CN/0.1% formic acid (the HPLC, mass spectrometer and columns were all from Thermo Fisher Scientific). The Orbitrap mass spectrometer was operated in the standard “top 15 or top 10” data-dependant acquisition modes while the preview mode was disabled. The MS full scan was set at *m*/*z* 380–1600 with the resolution at 30,000 at *m*/*z* 400 and AGC at 1 × 10^6^ with a maximum injection time at 200 msec. The 15, or 10, most abundant multiply-charged precursor ions, with a minimal signal above 3000 counts, were dynamically selected for CID fragmentation (MS/MS) in the ion trap, which had the AGC set at 5000 with the maximum injection time at 100 msec. The dynamic exclusion duration time was set for 60 s with ± 10 ppm exclusion mass width.

The raw files were processed in MaxQuant (Version 1.5.2.8, www.MaxQuant.org) for both protein identification and protein quantification. The *C. jejuni* M1 protein database was a combination of those downloaded from UniprotKB (www.uniprot.org) of 11,168 (April 2015) and M1 (February 2015). Parameters used were mainly in default values with some modifications: trypsin with maximum 2 missed cleavage sites, peptide mass tolerance at first search was set at 20 ppm and main search was at 4.5 ppm, MS/MS fragment mass tolerance at 0.50 Da, and top 8 MS/MS peaks per 100 Da and a minimum peptide length of 7 amino acids were required. Fixed modification for Carbamidomethyl and variable modification for Acetyl (Protein N-term), Deamidated (NQ) and Oxidation (M) were used. False discovery rates (FDR) were estimated based on matches to reversed sequences in the concatenated target-decoy database. The maximum FDR at 1% was allowed for proteins and peptide spectrum matches (PSMs). Peptides were assigned to protein groups, a cluster of a leading protein(s) plus additional proteins matching to a subset of the same peptides. For protein quantification, the minimum ratio of two, from ‘unique and razor peptides’ was required, and Re-quantify was enabled but Match between runs was disabled. The protein FDR was set to 0.1%. The MaxQuant output was processed using Perseus (Version 1.5.2.6 www.MaxQuant.org). Protein groups that are only identified by site, or reverse matches and potential contaminants. Are filtered out. A log_2_ transformation of SILAC ratio was carried out, rows were filtered to contain a minimum of two values and filtered using a Benjamini-Hochberg FDR test to 0.05. The SILAC data is provided in Table S2, the label-free LC/MS data is provided in Table S3.

### 2.5 Immunoblotting

Proteins were run on 4–12% SDS polyacrylamide gels, and subsequently transferred to PVDF membranes at 100 V for one hour. All blocking steps were carried out shaking at 30 rpm using 3% fat-free skimmed milk. Primary monoclonal anti-FLAG M2 antibody (Sigma) was used at a concentration of 1:1000. Secondary goat anti-mouse IgG-HRP (Santa Cruz Biotechnology) was used at a concentration of 1:5000. Primary and secondary antibodies were diluted in 3% fat-free skimmed milk. Washing steps were carried out with PBS containing 0.05% Tween 20, and bands were detected using Super Signal West Pico chemiluminescent substrate (Thermo Fisher Scientific).

### 2.6 Generation of defined gene deletion mutants and FLAG-tagged constructs

Construction of gene deletion mutants was carried out by allelic replacement as previously described [20], with each gene of interest being replaced with a chloramphenicol resistance cassette (*cat*). Primer sequences are provided in Table S4. The *cat* cassette was amplified from plasmid pCC027 [21] while 5′ and 3′ flanking regions for each gene of interest were amplified from *C. jejuni* strain M1 genomic DNA. Primers used to amplify the 5′ and 3′ flanking regions also contained overlapping sequence for the *cat* cassette allowing this to be inserted between the desired 5′ and 3′ sequences by primer-less PCR amplification to create a 5′ flank-*cat* cassette-3′ flank product. This product was used as a template to amplify the desired sequence for allelic replacement by electroporation, using the forward primer for 5′ flank amplification and the reverse primer for 3′ flank amplification. Electroporation and subsequent natural transformation was carried out as described previously [20], resulting in transformation of *C. jejuni* strain M1 and the generation of a coupled wild type strain.

FLAG-tagged proteins of interest were generated in *C. jejuni* M1 and *C. jejuni* 81–176 by allelic replacement using a kanamycin resistance cassette. Amplification of the kanamycin cassette was carried out using plasmid pRY107 [22] as a template, while flanking regions were amplified using as template genomic DNA from either *C. jejuni* M1 or *C. jejuni* 81–176, genomic DNA. For each FLAG-tagged construct, a 5′ flanking region was amplified consisting of ~ 400 bp upstream and an ORF of interest with the FLAG sequence incorporated immediately before the stop codon. The reverse primer for the 5′ flanking region also contained overlapping sequence with the kanamycin resistance cassette. The 3′ flanking region consisted of ~ 400 bp downstream of the ORF of interest with the forward primer containing an overlapping sequence with the kanamycin resistance cassette. As above for gene deletion, primer-less PCR amplification was carried out to combine a 5′ flank-kanamycin cassette-3′ flank product. This product, containing the kanamycin resistance cassette, was then used to PCR amplify the desired sequence for allelic replacement, by using the forward primer for the 5′ flank amplification and the reverse primer from the 3′ flank amplification. Electroporation and natural transformation for native FLAG-tag incorporation was carried out as described for the creation of defined gene deletions [20].

### 2.7 Motility assay

*C. jejuni* was grown on BHI blood agar plates for ~ 48 h then re-plated on BHI blood plates for ~ 16 h. Suspensions of *C. jejuni* scraped from plates into BHI were diluted to OD_600nm_ of ~ 0.5 and were used to stab motility plates comprised of BHI broth containing 0.4% select agar (Sigma). Motility plates were incubated for ~ 16 h, following which the diameter of the zone of motility was measured (n = 3).

### 2.8 Culture of CACO-2 cells

CACO-2 cell lines were purchased from the ATCC (CC-L244, HTB-37). Cells were grown using DMEM (Gibco) supplemented with 10% FBS and 1% non-essential amino acids. Cells were routinely grown in 75 cm^2^ tissue culture flasks and incubated at 37 °C with 5% CO_2_ in a humidified atmosphere.

### 2.9 CACO-2 cell infection assays

CACO-2 cells were seeded at 2 × 10^5^ cells on 12 well plates (Greiner) until confluency was observed. CACO-2 cells were infected with different *C. jejuni* strains at a multiplicity of infection of 100. To assay adherence/invasion, infected cells were incubated with 5% CO_2_ in a humidified atmosphere for 2 h. At this point non-adherent bacteria were removed, subjected to 10-fold serial dilutions and plated on BHI blood agar plates with 5 μg/ml TrM. Wells were washed three times with PBS, and cells were lysed with 0.1% Triton-X-100 in PBS for 15 min. Lysed cells were subjected to 10-fold serial dilutions and plated on BHI blood agar plates with 5 μg/ml TrM. To determine the number of internalized bacteria, infected CACO-2 cells were incubated at 37 °C with 5% CO_2_ in a humidified atmosphere. After 2 h, the medium overlaying the infected cells was changed to complete DMEM containing 250 μg/ml gentamycin sulphate and infected cells were incubated with 5% CO_2_ in a humidified atmosphere for a further 2 h. Cells were then washed three times with PBS and lysed with 0.1% Triton X-100 in PBS for 15 min. Serial dilutions of the cell lysates were carried out and plated on BHI blood agar plates with 5 μg/ml TrM. Dilutions of mutant *C. jejuni* strains were plated on BHI blood agar plates containing 10 μg/ml chloramphenicol. All plates were incubated for 48 h under microaerophilic conditions at 42 °C before colony counting took place. For both total association and invasion experiments, the percentage of *C. jejuni* interacting with CACO-2 cells was calculated as a percentage of the non-adherent fraction, to account for potentially different survival profiles of different strains in DMEM (n = 3).

## 3 Results

### 3.1 The *C. jejuni* M1 FT3SS-dependent secretome, as defined by quantitative proteomics

**Fig. 1:**
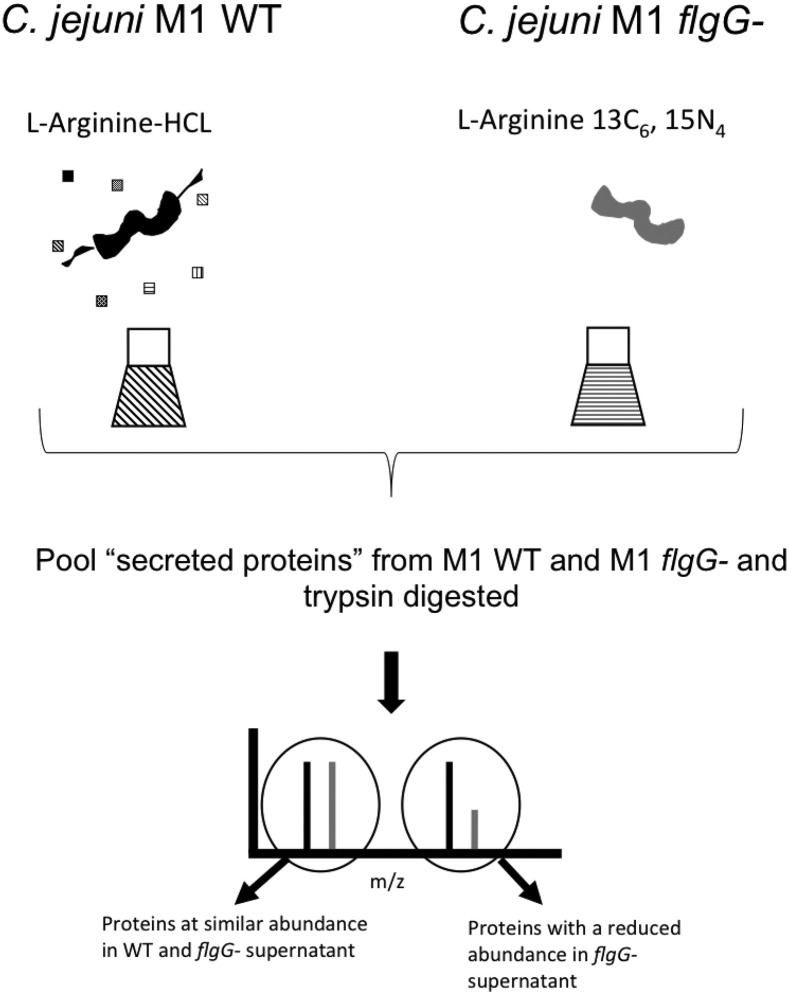
Overview of M1 secretome analysis. After isotopic incorporation of *C. jejuni* M1 WT and *flgG* strains with either “light” or “heavy” arginine, respectively, (H = mutant labelled with “heavy” l-arginine, L = WT labelled with “light” arginine), bacterial cells were pelleted and supernatants were filtered, pooled and concentrated. After digestion, tryptic peptides were analysed by LC-MS/MS. Proteins secreted *via* the flagellum are characterized by enrichment of peptides containing “light” isotopes.

**Fig. 2:**
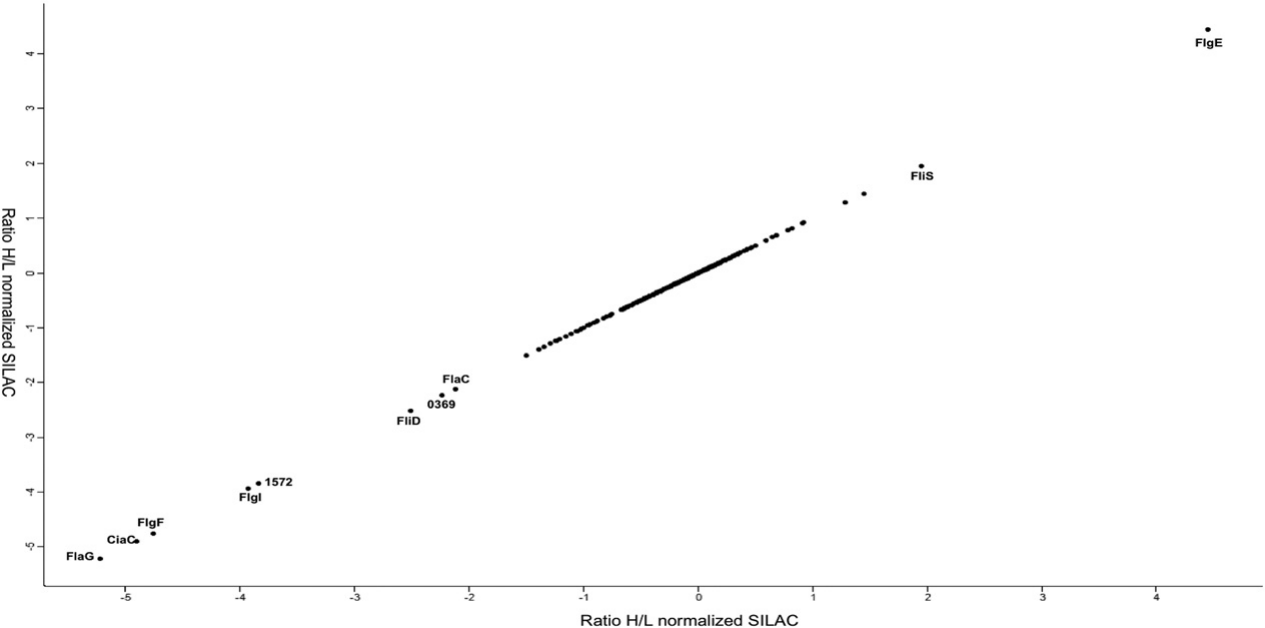
Mean H/L ratios of proteins detected in culture supernatants from four biological replicates. Low H/L ratios represent proteins found at a lower abundance within *flgG* supernatants while high H/L are proteins found at increased abundance within *flgG* supernatants.

We designed a quantitative proteomics experiment utilizing SILAC to screen proteins whose secretion from *C. jejuni* strain M1 may be mediated by the FT3SS (Fig. 1). To inhibit flagellar secretion an isogeneic M1 *flgG* mutant was generated. *C. jejuni* strains with a disrupted *flgG* gene are inhibited in their secretion of several proteins *via* the FT3SS [6]. The vast majority of proteins identified by SILAC analysis were similarly abundant in WT and *flgG* supernatants (Fig. 2), with similar observations having been described for *Escherichia coli* and *Salmonella enterica* SILAC secretome screens [23], [24]. Proteins possessing H/L ratios lower than − 2 (H = mutant labelled with “heavy” l-arginine, L = WT labelled with “light” l-arginine) from SILAC analysis are shown in Table 1. Unsurprisingly, these comprised proteins associated with flagellar gene regulation or structure (FlgM, FlaG), but also FlaC, CiaC, CJM_1572 and CJM1_0369. A number of proteins were found at an increased abundance within *flgG* supernatants. Proteins possessing the highest H/L ratios largely represented factors that accommodate correct flagellar assembly (Fig. 2). To increase the proteome coverage, and also account for potential incomplete incorporation of “heavy” or “light” arginine in the mutant or WT, proteomic analysis of unlabelled M1 *flgG* and WT supernatants was also performed. Proteins detected within the supernatant of M1 WT but not within *flgG* supernatants are shown in Table 2. From the SILAC and unlabelled LC/MS data, 5 proteins were chosen for further investigation (CiaI, FlaC, FspA, CJM1_0791 and CJM1_0395). These proteins represented non-flagellar proteins, none of which has been characterized in strain M1, that were present at contrasting abundance within WT and *flgG* supernatants.

### 3.2 FLAG-tagging of protein validates MS datasets

**Fig. 3:**
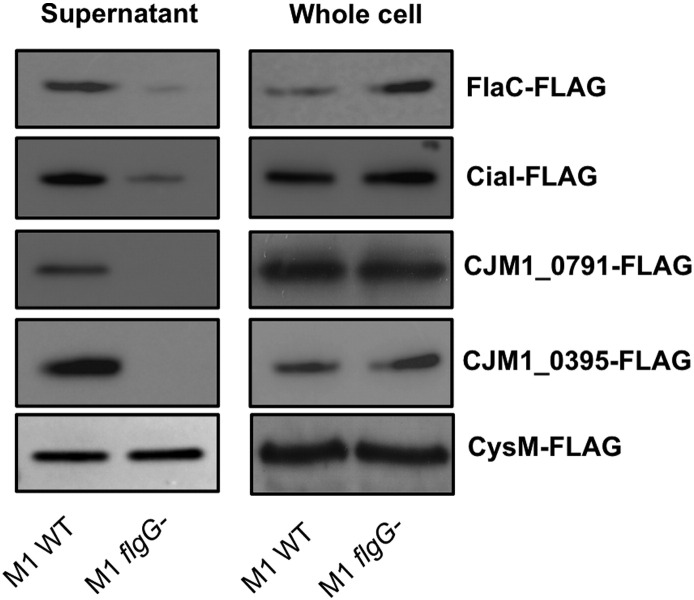
Western blotting of FLAG-tagged proteins secreted from M1 WT and *flgG* strains identified at various H/L ratios within the SILAC experiment. Detection of FLAG-tag incorporated into C-terminus of proteins of interest. Immunoblotting exhibits the effect of an *flgG* mutant on protein abundance within supernatant and whole cell protein samples.

A number of the proteins selected for further investigation were FLAG-tagged and assessed for their presence in supernatants obtained from M1 WT and *flgG* strains. As shown in Fig. 3, CiaI and FlaC were present in the concentrated supernatants from both M1 WT and *flgG*, although at a very reduced level from the *flgG* mutant. CJM1_0791 and CJM1_0395 were present only within M1 WT supernatants, confirming the data obtained by label-free LC/MS. Analysis of CysM was included for FLAG-tagging as it has previously been used as a marker for cell lysis [13]. Surprisingly CysM was readily detected within supernatant samples isolated from both M1 WT and *flgG* within the SILAC data.

### 3.3 Defined gene deletion mutants alter the interaction of *C. jejuni* with CACO-2 cells

**Fig. S1:**
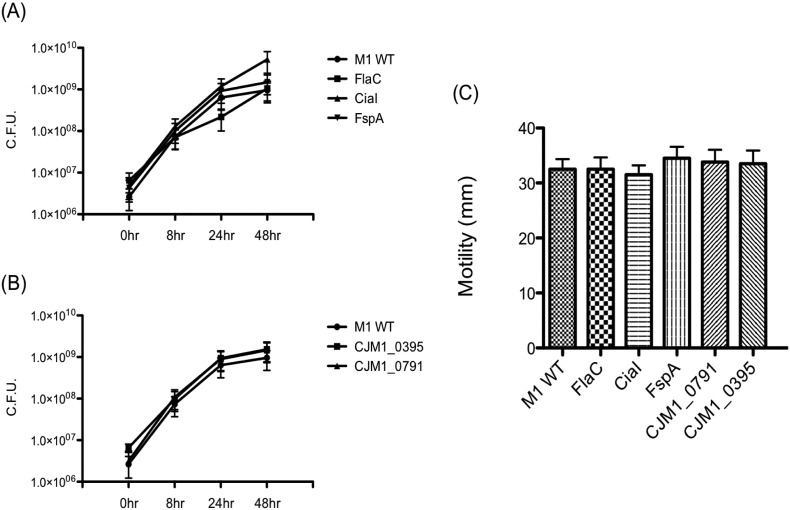
Generated M1 mutants display similar rates of growth and motility to that of M1 WT. (A, B) CFU counts of mutants generated, compared to M1 WT grown in MH broth. Cultures were equalized to an OD_600nm_ prior to incubation. (C) Motility of M1 mutants are comparable to that of M1 WT.

**Fig. 4:**
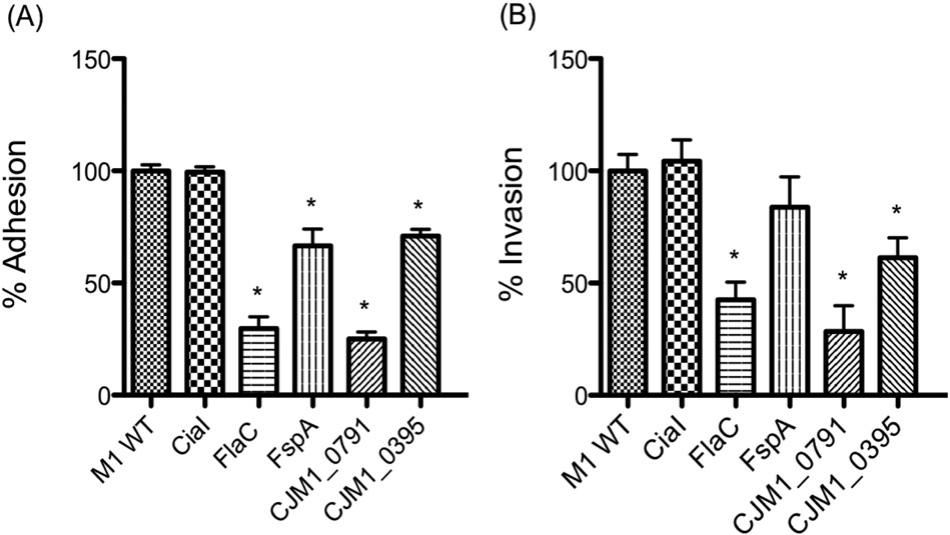
Mutations in genes previously characterized to be secreted *via* the flagellum and proteins identified by SILAC and label-free LC/MS have altered rates of (A) adhesion and (B) invasion of CACO-2 cells. M1 WT cell interaction was set at 100%, * denotes strains with a P value < 0.05 compared to M1 WT.

Individual isogenic deletion mutants of M1 were generated that lack *ciaI*, *flaC*, *fspA*, *CJM1*_*0791* and *CJM1*_*0395* were generated. The abilities of these mutants to interact with CACO-2 cells were measured. Rates of growth and motility for each of the mutants were consistent with that of the WT (Fig. S1). Fig. 4 shows the interaction for each strain with CACO-2 cells, represented as a percentage of the coupled M1 WT CACO-2 cell interaction. Each of the mutants, except *ciaI*, adhered less well than the WT. There was a statistically significant difference in invasion of each of the mutants except for *ciaI* and *fspA*. Disruption of M1 genes *flaC* and *CJM1_0791* resulted in more severe defects for adhesion and invasion than that observed for *fspA* and *CJM1_0395*.

### 3.4 *C. jejuni* strain 81–176 CJM1_0791 and CJM1_0395 genes are dependent on the flagellum for translocation to the supernatant

**Fig. 5:**
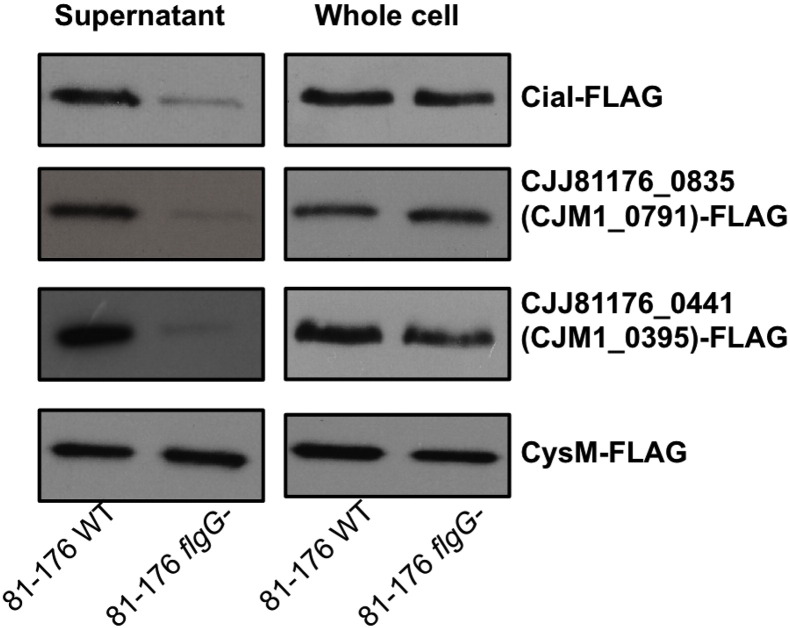
81–176 homologues of CJM1_0791 and CJM1_0395 are dependent on the flagellum for their presence in 81–176 supernatant. Immunoblotting of FLAG-tagged proteins exhibit the effect of an *flgG* mutant on protein abundance present in supernatant and whole cell protein samples obtained from strain 81–176.

To investigate whether the absence of CJM1_0791 (CJJ81176_0835) and CJM1_0395 (CJJ81776_0441) within M1 *flgG* supernatants was strain-specific, these proteins were FLAG-tagged in *C. jejuni* 81–176 WT and 81–176 *flgG* backgrounds. The CiaI and CysM protein equivalents were also FLAG-tagged. As shown in Fig. 5, CJJ81176_0441 (equivalent to CJM1_0395) was absent from the 81–176 *flgG* supernatant, consistent with data from M1. A very low level of CJJ81176_0835 (equivalent to CJM1_0791) was detected within the *flgG* supernatant, although there was a much higher abundance of the protein within the 81–176 WT supernatant. Furthermore, CiaI was present at a much higher level within the WT supernatant although still detectable within the *flgG* mutant supernatant, while CysM was readily detectible within the supernatants from both strains.

## 4 Discussion

The mechanisms by which *C. jejuni* invades human intestinal cell lines without a dedicated system for the secretion of effector proteins associated with virulence remains unknown, and is among the most important subjects for study regarding *C. jejuni* pathogenesis. Previous studies have made good progress in identifying the *C. jejuni* flagellum as an organelle that mediates extracellular secretion of non-flagellar proteins. Some of these secreted proteins have been proposed to contribute to the invasion of human cell lines by *C. jejuni* [5], [6], [7], [8], [9], [10], [11], [12], [13], [14], [15]. In this study we have used a combination of SILAC and label-free LC/MS to screen *C. jejuni* secretomes obtained from WT M1 and flagellum-deficient strains.

SILAC analysis measuring the relative abundances of proteins from M1 WT and *flgG* supernatants revealed that most proteins were present at a relatively equal abundance within supernatants from the WT and *flgG* mutant (Fig. 2). Similar observations have been made for SILAC secretome studies using *S. enterica* and *E. coli* in the past [23], [24]. Of the proteins that scored the lowest H/L ratios, the presence of CiaI and FlaC adds further support to existing evidence for their proposed utilisation of the flagellum as a mechanism of extracellular secretion [11], [15]. It was also observed that CiaI was secreted from M1 WT when analysed by label-free LC-MS. The role of the flagellum in CiaI and FlaC secretion was further confirmed by FLAG-tagged CiaI and FlaC being present at reduced levels (as detected by Western blotting using an antibody against the FLAG-tag) in *flgG* mutant supernatants (Fig. 3 and Fig. 5). The reduced abundance of FlaC in *flgG* supernatants is in agreement with the M1 SILAC data; this has not been observed in previous studies [11], [15]. Infection assays revealed no apparent influence of CiaI on the ability of M1 to adhere to or invade CACO-2 cells. Disruption of *flaC* appeared to reduce levels of both M1 adherence and invasion of CACO-2 cells (Fig. 4). For CiaI this is at odds with what has been described regarding invasion using *ciaI* mutants in strains 81–176 and F38011 [11], [12]. It may be that either M1 *ciaI* does not display the same phenotype as in 81–176 or F38011, or that a reduced interaction for any *C. jejuni ciaI* mutant does not occur for CACO-2 cells as it does for T84 cells. Previously it has been described that *flaC* disruption reduces Hep-2 cell invasion by *C. jejuni* TGH9011 but does not alter adherence [15], as it does in M1. Further work is required to establish the function of FlaC across multiple *C. jejuni* strains, and its role during *C. jejuni* pathogenesis. It is also notable that neither CiaC (CJM1_1224) nor CiaD (CJM1_0764) were detected in the SILAC data set. However, both proteins were detected in both WT and *flgG* supernatants in the label-free LC/MS data set. Detection of proteins within the LC/MS but not the SILAC dataset may represent an incomplete incorporation of exogenous arginine labelling, as the external addition of “heavy” or “light” arginine is the only difference between media used for the SILAC and label-free LC/MS experiments and highlights the importance of unlabelled LC/MS to account for this possibility. This may also account for the lack of CiaD detection within the SILAC dataset as the protein contains a single arginine. Previous work has shown that Cia expression and secretion is increased in the presence of external stimuli [25], [26]. An excellent future use of SILAC would be to compare supernatants isolated from bacteria in the presence or absence of a stimulus such as bile salts. This would enable a quantitative measurement of the effect of stimuli on Cia secretion, and may facilitate the identification of further proteins, part of the Cia family or otherwise, which contribute to *C. jejuni* human tissue culture cell interaction.

CJM1_0791 and CJM1_0395 were chosen for further investigation. Immunoblotting of each of these FLAG-tagged proteins reflected the levels observed in either the SILAC or label-free LC/MS data sets.

The dependence of CJM1_0791 and CJM1_0395 on the flagellum for their presence in the supernatant is to our knowledge the first time that this has been reported. CJM1_0791 is a putative periplasmic lipoprotein. Disruption of *CJM1_0791* led to a reduction in both rates of adhesion and invasion (Fig. 4). The decreased adherence of the *CJM1_0791* mutant, its status as a putative lipoprotein, and its relatively large molecular weight (48.9 kDa), make it possible that its absence within *flgG* supernatants is an indirect effect of knocking out the flagellum. The disruption of a large trans-membrane bound organelle, such as the flagellum, could feasibly lead to membrane structural alteration and a reduction in the presence of other proteins predicted to be membrane bound such as CJM1_0791. Further studies will be necessary to observe whether the protein actually passes through the flagellum, or to identify what secondary effects resulting from flagellar disruption result in its absence from the supernatant. In fact, it was noted that a small number of proteins, which one would not expect to be secreted, were consistently reduced in their abundance within the SILAC dataset from the M1 *flgG* supernatants (Fig. 2). Similar findings have occurred when using SILAC to study the secretomes of other bacteria, such as the ribosomal protein L21 being present in the SPI-2 (*Salmonella* Pathogenicity Island 2) dependent secretome of *S. enterica* [23]. The presence of CysM within both WT and *flgG* supernatants was a surprising observation considering its use as a marker for cell lysis in the past [13]. It is clear from the data shown here that CysM is found within M1 and 81–176 supernatants, although the reasoning for this is unclear. Future studies will be needed to determine whether CysM is found within the supernatants of more *C. jejuni* strains, as has been observed here for M1 and 81–176. CJM1_0395 (26 kDa) is a member of the SprA-related superfamily of zinc metalloproteases, and represents a good candidate as a novel non-flagellar protein secreted *via* the FT3SS. Another zinc metalloprotease, NleC, present in *E. coli* and *S. enterica* and exported *via* the T3SS also does not have a large effect on rates of epithelial cell invasion as observed for a *CJM1_0395* mutant, but impairs an NF-kB mediated inflammatory response during infection [27], [28], [29]. Although there is no sequence similarity between the two proteins, future work will be required to address whether CJM1_0395 is delivered to host cells, and whether it may contribute to aspects of *C. jejuni* disease other than a direct influence on rates of adhesion/invasion of *in vitro* grown tissue culture cells.

In conclusion, in this study we have applied quantitative proteomics to study the *C. jejuni* flagellum-dependent secretome quantitatively for the first time. The combination of SILAC and unlabelled proteomics has led to the identification of two proteins, CJM1_0791 and CJM1_0395, which appear dependent on a WT flagellum for their presence within *C. jejuni* supernatant, representing putative effector proteins that regulate infection. We have also further defined the influence of the flagellum on CiaI and FlaC secretion for *C. jejuni* strain M1, and identified novel genes that alter the ability of M1 to interact with CACO-2 cells. Future work combining the use of existing and developing proteomic technologies will allow for a significantly more in-depth assessment of *C. jejuni* biology and host-pathogen interactions.

The following are the supplementary data related to this article.

Supplementary material Image 1

Table S2 SILAC data of H/L ratios from 4 biological replicates generated from M1 WT and M1 *flgG* supernatants. Table S2

Table S3 Label-free LC/MS data from M1 WT and M1 *flgG* supernatants. Table S3

## Conflict of interest

The authors declare that there is no conflict of interest.

## Figures and Tables

**Table 1:** Proteins with the H/L ratios below − 2 (H = labelled mutant, L = labelled WT) from the SILAC screen. Values included are the mean from four biological replicates.

Ratio H/L normalized	Description
− 5.22125	FlaG
− 4.90379	CiaC
− 4.76184	FlgF
− 3.93479	FlgI
− 3.83989	CJM1_1572
− 2.514	FliD
− 2.23358	CJM1_0369
− 2.11747	FlaC

**Table 2:** Proteins identified within M1 WT supernatants that were absent in M1 *flgG* mutant supernatants as detected by standard LC/MS.

Razor + unique peptides WT	Razor + unique peptides *flgG*	Q-value	Description
9	0	0	Uncharacterized protein CJM1_1572
8	0	0	Uncharacterized protein CJM1_0791
7	0	0	CiaI
6	0	0	FspA
4	0	0	Uncharacterized protein CJM1_0395
3	0	0	Uncharacterized protein CJM1_0821
3	0	0	Uncharacterized protein CJM1_1598
2	0	0	PurD
2	0	0	MutS
2	0	0	RplT
2	0	0	CmeE
2	0	0	IspH
2	0	0	FlgG
2	0	0	FtsZ
2	0	0	PseA
2	0	0	FliE
2	0	0	FlgB
