# Supplementary material for: A quantitative proteomic screen of the Campylobacter jejuni flagellar-dependent secretome
Source: J Proteomics. 2017 Jan 30;152:181–7. doi: 10.1016/j.jprot.2016.11.009 (PMC5223770; doi:10.1016/j.jprot.2016.11.009)
Supplement: Supplementary file 1 — Supplementary material [file mmc1.docx]

**SUPPLEMENTAL INFORMATION**

**FIGURE AND TABLE LEGENDS**

**Fig. S1.** Generated M1 mutants display similar rates of growth and motility to that of M1 WT. (A, B) CFU counts of mutants generated, compared to M1 WT grown in MH broth. Cultures were equalized to an OD_600nm_ prior to incubation. (C) Motility of M1 mutants are comparable to that of M1 WT.

**Table S1.** Bacterial strains used in this study.

**Table S2.** SILAC data of H/L ratios from 4 biological replicates generated from M1 WT and M1 *flgG* supernatants.

**Table S3.** Label-free LC/MS data from M1 WT and M1 *flgG* supernatants.

**Table S4.** Oligonucleotides used in this study.

**Supplemental references**

| **Strain** | **Genotype** | **Source/Reference** |
| --- | --- | --- |
| *C. jejuni* M1 | Wild-type | Diane Newell, (S1) |
| *C. jejuni* 81-176 | Wild-type | (S2) |
| M1 Δ*flgG* | Δ*flgG*;Cm^r^ | This study |
| M1 Δ*ciaI* | Δ*ciaI*;Cm^r^ | This study |
| M1 Δ*flaC* | Δ*flaC*;Cm^r^ | This study |
| M1 Δ*fspA* | Δ*fspA*;Cm^r^ | This study |
| M1 Δ*CJM1_0791* | Δ*CJM1_0791*;Cm^r^ | This study |
| M1 Δ*CJM1_0395* | Δ*CJM1_0395*;Cm^r^ | This study |
| M1 CiaI-FLAG | *ciaI*-FLAG;Km^r^ | This study |
| M1 FlaC-FLAG | *flaC*-FLAG;Km^r^ | This study |
| M1 CJM1_0791-FLAG | *CJM1_0791-*FLAG;Km^r^ | This study |
| M1 CJM1_0395-FLAG | *CJM1_0395-*FLAG;Km^r^ | This study |
| M1 CysM-FLAG | *cysM*-FLAG;Km^r^ | This study |
| M1 Δ*flgG,* CiaI-FLAG | Δ*flgG, ciaI*-FLAG;Cm^r^,Km^r^ | This study |
| M1 Δ*flgG,* FlaC-FLAG | Δ*flgG, flaC*-FLAG;Cm^r^,Km^r^ | This study |
| M1 Δ*flgG,* CJM1_0791-FLAG | Δ*flgG, CJM1_0791*-FLAG;Cm^r^,Km^r^ | This study |
| M1 Δ*flgG,*CJM1_0395-FLAG | Δ*flgG, CJM1_0395*-FLAG;Cm^r^,Km^r^ | This study |
| M1 Δ*flgG,* CysM-FLAG | Δ*flgG, cysM*-FLAG;Cm^r^,Km^r^ | This study |
| 81-176 CiaI-FLAG | *ciaI*-FLAG;Km^r^ | This study |
| 81-176 CJJ81176_0835-FLAG | *CJJ81176_0835*-FLAG;Km^r^ | This study |
| 81-176 CJJ81176_0441-FLAG | *CJJ81176_0441*-FLAG;Km^r^ | This study |
| 81-176 CysM-FLAG | *cysM*-FLAG;Km^r^ | This study |
| 81-176 Δ*flgG* CiaI-FLAG | Δ*flgG*, *ciaI*-FLAG;Cm^r^, Km^r^ | This study |
| 81-176 Δ*flgG* CJJ81176_0835-FLAG | Δ*flgG*, *CJJ81176_0835*-FLAG;Cm^r^,Km^r^ | This study |
| 81-176 Δ*flgG* CJJ81176_0441-FLAG | Δ*flgG*, *CJJ81176_0441*-FLAG;Cm^r^,Km^r^ | This study |
| 81-176 Δ*flgG* CysM-FLAG | *ΔflgG, cysM*-FLAG;Cm^r^,Km^r^ | This study |

**Table S1.**

| **Primer name** | **Description** | **Sequence 5’ to 3’** |
| --- | --- | --- |
| L_WITS_1_cam_FW1 | Amplification of Cm^r^ cassette | AAGCTTGGCGTGGGAGTG |
| R_WITScam_RV1 | Amplification of Cm^r^ cassette | GAATTCCGCCCTTTAGTTCC |
| CC069 | Presence of Cm^r^ cassette | ATATGTGCAGGGCGTATTGC |
| FlgG_L1_FW1 | Amplification of left flank for *flgG* mutant creation | GATGATATAAGAGCTTTGCAAA |
| FlgG_L2_RV1 | Amplification of left flank for *flgG* mutant creation | CTCACTCCCACGCCAAGCTTCTGCAAGCTTATTGATAGCTTC |
| FlgG_R1_FW1 | Amplification of right flank for *flgG* mutant creation | GGAACTAAAGGGCGGAATTCTAGGAATTGTAAATCAGCTTAA |
| FlgG_R2_RV1 | Amplification of right flank for *flgG* mutant creation | TCAAAACAGATCAGCAAGTTGT |
| FlgG_A_RV1 | Control for *flgG* allelic replacement | CATCAATTTGTGTTTGCTGCGC |
| FlaC_L1_FW1 | Amplification of left flank for *flaC* mutant creation | AGGAGAAGGAGTGTTGAGCC |
| FlaC_L1_RV1 | Amplification of left flank for *flaC* mutant creation | CTCACTCCCACGCCAAGCTTTCCACTTATTGCGCGAACAG |
| FlaC_R1_FW1 | Amplification of right flank for *flaC* mutant creation | GGAACTAAAGGGCGGAATTCGCAATCCAACATGCAGCTTC |
| FlaC_R2_RV1 | Amplification of right flank for *flaC* mutant creation | TTGGATCTAGACCGCTTTTG |
| FlaC_A_RV1 | Control for *flaC* allelic replacement | TTCATTAAGTCTATCTGCGC |
| CiaI_L1_FW1 | Amplification of left flank for *ciaI* mutant creation | TGCCATTTTAAACTCGGCCT |
| CiaI_L1_RV1 | Amplification of left flank for *ciaI* mutant creation | CTCACTCCCACGCCAAGCTTTGCATAACTTACGCTTTGATTGT |
| CiaI_R1_FW1 | Amplification of right flank for *ciaI* mutant creation | GGAACTAAAGGGCGGAATTCAGCCAAGGAAAATCAAGATAAGA |
| CiaI_R2_RV1 | Amplification of right flank for *ciaI* mutant creation | AGGATGCTCTTCTTCTTTACAA |
| CiaI_A_RV1 | Control for *ciaI* allelic replacement | TGTGTGAGCTTAGAGCCTTC |
| FspA_L1_FW1 | Amplification of left flank for *fspA* mutant creation | TGTGTTAGCAAAAGCCCAAAA |
| FspA_L1_RV1 | Amplification of left flank for *fspA* mutant creation | CTCACTCCCACGCCAAGCTTTACAGCTGGATCTTGCGCTA |
| FspA_R1_FW1 | Amplification of right flank for *fspA* mutant creation | GGAACTAAAGGGCGGAATTCACTGCGAAAATGTCTAAAGCT |
| FspA_R2_RV1 | Amplification of right flank for *fspA* mutant creation | TGAAAATTGACCTTAGCGCCT |
| FspA_A_RV1 | Control for *fspA* allelic replacement | AAGCTTGAGCTGGAGCTTGA |
| 0395_L1_FW1 | Amplification of left flank for *CJM1_0395* mutant creation | CTCTTTGCTGTTTTCCCCACA |
| 0395_L1_RV1 | Amplification of left flank for *CJM1_0395* mutant creation | CTCACTCCCACGCCAAGCTTCCATTTGGGTTTGTTCGCCA |
| 0395_R1_FW1 | Amplification of right flank for *CJM1_0395* mutant creation | GGAACTAAAGGGCGGAATTCAGCCGAAGAAGCCAAAGAAA |
| 0395_R2_RV1 | Amplification of right flank for *CJM1_0395* mutant creation | CTCTTGGAGGGTGGTATGCA |
| 0395_A_RV1 | Control for *CJM1_0395* allelic replacement | GCTGCGATTTGTCTTGCATT |
| 0791_L1_FW1 | Amplification of left flank for *CJM1_0791* mutant creation | GTGCTTTGCGATAAAGAAGGGTA |
| 0791_L1_RV1 | Amplification of left flank for *CJM1_0791* mutant creation | CTCACTCCCACGCCAAGCTTCTTTATCTACACTATAACCATAGGC |
| 0791_R1_FW1 | Amplification of right flank for *CJM1_0791* mutant creation | GGAACTAAAGGGCGGAATTCAATTCCTACGCTGATTCTATAATGG |
| 0791_R2_RV1 | Amplification of right flank for *CJM1_0791* mutant creation | AACCGCATATAAATGTCCTGTAG |
| 0791_A_RV1 | Control for *CJM1_0791* allelic replacement | CATTATATTCAGCCACTCTCTCTAT |
| Kan_FW1 | Amplification of Km^r^ cassette | TTGTTATAATTAGCTTCTTGGGGTA |
| Kan_RV1 | Amplification of Km^r^ cassette | CTAAAACAATTCATCCAGTAAAATATAATATTTTA |
| Kan_C_RV1 | Presence of Km^r^ cassette | GGAGTGTCTTCTTCCCAGTT |
| FlaC_L_F_TAG_FW1 | Amplification of left flank for *flaC* FLAG integration | TAGGAATAGTTGTTGCACCAACAAT |
| FlaC_L_R_TAG_RV1 | Amplification of left flank for  *flaC* FLAG integration | TACCCCAAGAAGCTAATTATAACAATTATTTATCATCATCATCTTTATAATCTTGTAATAAATTAGCAATTTTGCTT |
| FlaC_R_F_TAG_FW1 | Amplification of right flank for  *flaC* FLAG integration | TATTTTACTGGATGAATTGTTTTAGTATAAGCCCTAAATAGGGCTTATTT |
| FlaC_R_R_TAG_RV1 | Amplification of right flank for  *flaC* FLAG integration | TCATTACTCATCCCCATAGAGCAAA |
| FlaC_C_FW1 | Control for  *flaC* allelic replacement | TTTATTACAAGATTATAAAGATGATGATGATAAA |
| CiaI_L_F_TAG_FW1 | Amplification of left flank for *ciaI* FLAG integration | GCGTGAATTACATTGAACATTTATT |
| CiaI_L_R_TAG_RV1 | Amplification of left flank for *ciaI* FLAG integration | TACCCCAAGAAGCTAATTATAACAATTATTTATCATCATCATCTTTATAATCAGCGTAAAGATTTAAACTATCATCT |
| CiaI_R_F_TAG_FW1 | Amplification of right flank for *ciaI* FLAG integration | TATTTTACTGGATGAATTGTTTTAGATCATATAATATTAAAAATTATCAA |
| CiaI_R_R_TAG_RV1 | Amplification of right flank for *ciaI* FLAG integration | AAAGTAATTCTCCAAGATTAAATCC |
| CiaI_C_FW1 | Control for *ciaI* allelic replacement | ATCTTTACGCTGATTATAAAGATGATGATGATAAA |
| 0395_L_F_TAG_FW1 | Amplification of left flank for *CJM1_0395* FLAG integration | GTACTTAACAAAGACACCGC |
| 0395_L_R_TAG_RV1 | Amplification of left flank for *ciaI* CJM1_0395 integration | TACCCCAAGAAGCTAATTATAACAATTATTTATCATCATCATCTTTATAATCAGATGCGATATTGAATTTAATGTCA |
| 0395_R_F_TAG_FW1 | Amplification of right flank for *ciaI* CJM1_0395 integration | TATTTTACTGGATGAATTGTTTTAGAATTCTTAGATTTAAATAAAAAATT |
| 0395_R_R_TAG_RV1 | Amplification of right flank for *CJM1_0395* FLAG integration | AGCTTAGCTTGATAATAACTTGAGA |
| 0395_C_FW1 | Control for *CJM1_0395* allelic replacement | TATCGCATCTGATTATAAAGATGATGATGATAAA |
| 0791_L_F_TAG_FW1 | Amplification of left flank for *CJM1_0791*FLAG integration | GATGATGAGATTATTATCAATGTCCAA |
| 0791_L_R_TAG_RV1 | Amplification of left flank for *ciaI* CJM1_0791 integration | TACCCCAAGAAGCTAATTATAACAACTATTTATCATCATCATCTTTATAATCAACCCTAGTTTGTCCCAAAAGATTA |
| 0791_R_F_TAG_FW1 | Amplification of right flank for *ciaI* CJM1_0791 integration | TATTTTACTGGATGAATTGTTTTAGAAGTTTTAATTTTTTAGTTTGAAA |
| 0791_R_R_TAG_RV1 | Amplification of right flank for *CJM1_0791* FLAG integration | CTTACCTTGAATCACCTCCAAAATA |
| 0791_C_FW1 | Control for *CJM1_0791* allelic replacement | AACTAGGGTTGATTATAAAGATGATGATGATAAA |

**Table S4.**

**Supplemental references**

1. C. Friis, T.M. Wassenaar, M.A. Javed, L. Snipen, K. Lagesen, P.F. Hallin, D.G. Newell, M. Toszeghy, A. Ridley, G. Manning, D.W. Ussery, Genomic Characterization of *Campylobacter jejuni* Strain M1, PLoS. One. 5 (2010) e12253.
2. J.A. Korlath, M.T. Osterholm, L.A. Judy, J.C. Forfang, R.A. Robinson, A Point-Source Outbreak of Campylobacteriosis Associated with Consumption of Raw Milk, J. Infect. Dis. 152 (1985) 592-596.
